# Dexamethasone treatment alters insulin, leptin, and adiponectin levels in male mice as observed in DIO but does not lead to alterations of metabolic phenotypes in the offspring

**DOI:** 10.1007/s00335-015-9616-5

**Published:** 2015-12-11

**Authors:** Clemens Bönisch, Martin Irmler, Laura Brachthäuser, Frauke Neff, Mareike T. Bamberger, Susan Marschall, Martin Hrabě de Angelis, Johannes Beckers

**Affiliations:** Institute of Experimental Genetics and German Mouse Clinic, Helmholtz Zentrum München GmbH - German Research Center for Environmental Health, Ingolstädter Landstr. 1, 85764 Neuherberg, Germany; Institute of Pathology, Helmholtz Zentrum München GmbH - German Research Center for Environmental Health, 85764 Neuherberg, Germany; Chair of Experimental Genetics, Technische Universität München, 85354 Freising, Germany; German Center for Diabetes Research (DZD), 85764 Neuherberg, Germany

## Abstract

Epigenetic inheritance (EI) of metabolic phenotypes via the paternal lineage has been shown in rodent models of diet-induced obesity (DIO). However, the factors involved in soma-to-germline information transfer remain elusive. Here, we address the role of alterations in insulin, leptin, and adiponectin levels for EI of metabolic phenotypes by treating C57BL/6NTac male mice (F0) with the synthetic glucocorticoid dexamethasone and generating offspring (F1) either by in vitro fertilization or by natural fecundation. Dexamethasone treatment slightly alters F0 body composition by increasing fat mass and decreasing lean mass, and significantly improves glucose tolerance. Moreover, it increases insulin and leptin levels and reduces adiponectin levels in F0 fathers as observed in mouse models of DIO. However, these paternal changes of metabolic hormones do not alter metabolic parameters, such as body weight, body composition and glucose homeostasis in male and female F1 mice even when these are challenged with a high-fat diet. Accordingly, sperm transcriptomes are not altered by dexamethasone treatment. Our results suggest that neither increased glucocorticoid, insulin, and leptin levels, nor decreased adiponectin levels in fathers are sufficient to confer soma-to-germline information transfer in EI of obesity via the paternal lineage.

**Electronic supplementary material:**

The online version of this article (doi:10.1007/s00335-015-9616-5) contains supplementary material, which is available to authorized users.

## Introduction

In the past decades, metabolic disorders (MDs) such as obesity, metabolic syndrome (MetS), and type 2 diabetes (T2D) became worldwide epidemics with continuously rising prevalence (Farag and Gaballa 2011; Kaur 2014). This development is mainly due to an increasing imbalance of energy intake and expenditure caused by changed environmental factors such as the growing availability of high-caloric diets and a sedentary lifestyle (Farag and Gaballa 2011; Kaur 2014). However, MDs also have a heritable component. Although many genetic variants that are linked to MDs have been identified, they commonly have small effect sizes and explain only a small percentage of heritability. For example, about 70 loci identified by genome-wide association studies (GWAS) account for only about 10 % of the heritability of T2D [reviewed in (Hara et al. 2014; Hirschhorn and Gajdos 2011)]. Also, a recent GWAS using body mass index (BMI) as a measure for obesity reported 97 loci that account for only 2.7 % of BMI phenotypic variation (Locke et al. 2015). The list of common and rare genetic variants that contribute to MD susceptibility is not complete, yet it is highly unlikely that genetic drift significantly contributed to the epidemic spread of diabetes in the past few decades (Stoger 2008). Instead, epidemiological and experimental studies point toward epigenetic inheritance (EI) as an important contribution to the observed epidemic spread of MDs (Desai et al. 2015; Rando and Simmons 2015; Somer and Thummel 2014; Stoger 2008; Szyf 2015). Importantly, EI could propagate responses to environmental stimuli much faster than genetic drift (Jablonka and Raz 2009).

Animal studies provided evidence for the contribution of both parents to EI. However, contribution from the maternal lineage is difficult to correlate to germline alterations due to the altered in utero environment during pregnancy and the close interaction between mother and offspring during lactation, which also affect the offspring’s metabolic status (Desai et al. 2015; Rando and Simmons 2015; Williams et al. 2014). Therefore, especially the contribution of the paternal lineage was in the focus in recent epigenetic studies as it is more confined to the germline. Studies in rodent models have shown that paternal diet-induced obesity (DIO) leads to alterations in body weight and glucose metabolism in the offspring (Fullston et al. 2013; Ng et al. 2010, 2014; Wei et al. 2014). Some of the studies also provided initial evidence for alterations in sperm that could explain the offspring´s phenotype, including changes in DNA methylation and miRNA content (Fullston et al. 2013; Ng et al. 2010; Wei et al. 2014). However, the mechanisms by which the paternal diet leads to germline alterations remain elusive, but a variety of possible triggers of soma-to-germline information transfer have been proposed. These include, in particular, inflammation (Fullston et al. 2013), hormones, and circulating RNAs (Jablonka and Raz 2009; Sharma 2013; Szyf 2015). Recently, one seminal study on the EI of altered hepatic wound-healing response provided evidence for the key role of factors present in plasma for the programming of epigenetic patterns in sperm (Zeybel et al. 2012); however, specific paternal factors were not identified.

In obese and pre-diabetic humans as well as in DIO animal models, plasma factors, including several hormones, are altered. Elevated levels of insulin (hyperinsulinemia) and leptin (hyperleptinemia) have been reported (Bluher 2014; Fullston et al. 2013; Kaur 2014; Ng et al. 2010; Rabe et al. 2008; Shimizu et al. 2007; Slieker et al. 1996; Wei et al. 2014). In addition, adiponectin levels are reduced (Bluher 2014; Kaur 2014), resulting in an increased leptin/adiponectin ratio, which serves as a robust biomarker for the metabolic syndrome (Falahi et al. 2013; Satoh et al. 2004). Importantly, the receptors for all three hormones are expressed in testis (Ahn et al. 2013; Ramachandran et al. 2013; Zamorano et al. 1997), providing a potential role of these hormones in soma-to-germline information transfer. Moreover, leptin receptor and proteins of the insulin signaling pathway are also expressed in sperm (Wang et al. 2013), potentially enabling direct signaling of both insulin and leptin to male gametes. Besides spermatozoa, semen also consists of seminal fluid-containing various factors important for fertilization, including hormones and cytokines. The potential involvement of seminal fluid in EI has been discussed (Bromfield 2014; Rando 2012) and, only recently, the absence of seminal fluid during mating has been shown to cause obesity and glucose intolerance in male offspring (Bromfield et al. 2014). This is consistent with the notion that alterations in seminal fluid contribute to non-genetic inheritance of metabolic phenotypes. Interestingly, adiponectin (Heinz et al. 2015), insulin, and leptin (Binder et al. 2015) are all present in seminal fluid. Moreover, the levels of insulin and leptin are increased in obese mice (Binder et al. 2015), making these hormones potential carriers of non-genetic intergenerational information by a mechanism independent of sperm.

In this study, we established a mouse model to address the role of the hormones insulin, leptin, and adiponectin for the intergenerational EI of metabolic phenotypes in the absence of obesity and most of its associated complications, such as low grade inflammation. Focusing on paternal contribution, we treated C57BL/6NTac male mice with dexamethasone, a synthetic glucocorticoid previously shown to increase insulin (Rafacho et al. 2014) and leptin (Slieker et al. 1996) levels and to decrease adiponectin levels (Fasshauer et al. 2002). We analyzed offspring generated via either in vitro fertilization (IVF) or natural matings with regard to obesity and glucose metabolism. The findings presented here make insulin, leptin, and adiponectin unlikely to be the major factors for EI reported in DIO rodent models before (Fullston et al. 2013; Ng et al. 2010, 2014; Wei et al. 2014). Accordingly, transcriptome analyses of sperm from dexamethasone-treated mice did not show significant alterations in gene regulation compared to control mice.

## Materials and methods

### Animals and diets

All mice (C57BL/6NTac) were housed under standard conditions (12:12 h light–dark cycle, light phase: 06:00–18:00 h). All animals of the F0 generation were fed a standard chow diet (Diet #1310, Altromin, Germany). All animals of the F1 generation were fed a standard chow diet until they were challenged with high-fat research diet (HFD) with 60 % energy from fat (EF R/M D12492 mod., Ssniff, Germany) starting at 9 weeks of age. All animal experiments were approved by the authorities of Upper Bavaria (Regierung von Oberbayern).

### Dexamethasone treatment (F0 generation) and phenotypic characterization

Starting at 9 weeks of age, male mice (F0) were either treated with dexamethasone (Dex) at a concentration of 2 mg/kg body weight (D4902, Sigma-Aldrich, USA) dissolved in (2-Hydroxypropyl)-β-cyclodextrin solution (H5784, Sigma-Aldrich, USA) at 10 mg/ml and further diluted 50-fold in sterile saline (final dexamethasone concentration 0.2 mg/ml) or were treated with vehicle (Veh), (2-Hydroxypropyl)-β-cyclodextrin solution 50-fold diluted in sterile saline. Every mouse received a total of 15 injections within 33 days (always on Monday, Wednesday, and Friday). During the treatment, body weight and blood glucose were measured once a week at 14:00 h after 6 h fasting. Body composition (MiniSpec LF50, Bruker Optics, Germany) was measured on the last day of the treatment (before the injection), and an intraperitoneal (ip) glucose tolerance test (GTT; 2 g/kg body weight glucose, Braun, Germany) was conducted 1 day after the treatment at 14:00 h (6 h fasted).

**Fig. 1:**
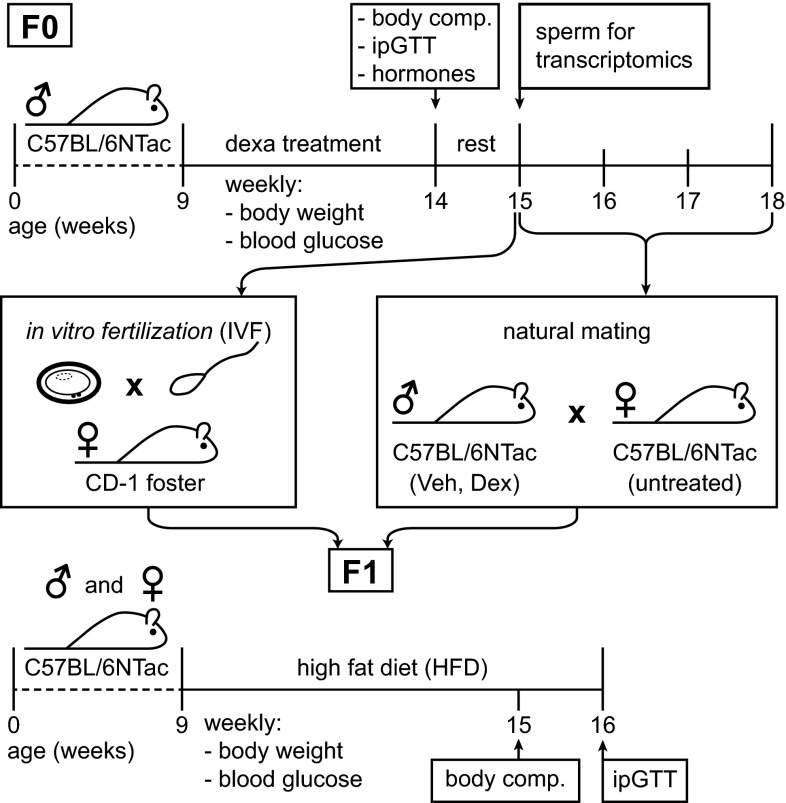
Study design. To analyze the role of insulin, leptin, and adiponectin levels in EI of metabolic phenotypes, we treated C57BL/6NTac male mice (F0) for 5 weeks with dexamethasone, starting at 9 weeks of age. Animals were tested for metabolic parameters as indicated (body weight, fasting blood glucose, hormone levels, body composition, and ipGTT). After 1 week of rest, sperm was obtained to generate offspring (F1) by IVF using oocytes from untreated C57BL/6NTac mice and transferring embryos into CD-1 foster mothers. Alternatively, males were mated with untreated C57BL/6NTac females. Male and female mice of the F1 generation were challenged with HFD starting at 9 weeks of age and metabolically analyzed as indicated

After 1 week of rest, animals were either mated with untreated females or sacrificed to collect organs and sperm, used either for in vitro fertilization (IVF) or for transcriptome analyses. In total, 19 males (9 Veh, 10 Dex) were used to generate offspring; 3 Veh versus 3 Dex was used in IVF; 6 Veh versus 7 Dex went into natural matings. For natural matings, every male was mated three times with two females, each time from Monday morning to Friday afternoon and single housed in between matings. For the F1 generation, starting with the beginning of the HFD challenge at 9 weeks of age, body weight and blood glucose were measured. Body composition was measured after 6 weeks of HFD challenge and an ipGTT was conducted after 7 weeks of HFD challenge. A scheme of the experimental layout is shown in Fig. 1.

### Plasma hormone levels and assessment of insulin resistance (HOMA-IR)

Directly before the start of the ipGTT at 14:00 h (6 h fasted), blood was collected from the tail tip into EDTA-coated tubes, centrifuged for 5 min at 1700×*g* (10 °C), and plasma aliquots were frozen in liquid nitrogen. Insulin and leptin were measured in parallel using the Mouse Metabolic Kit (MSD, USA) and adiponectin was measured using the Mouse Adiponectin Kit (MSD, USA) according to the manufacturer’s protocol.

Insulin resistance was determined by homeostatic model assessment-insulin resistance (HOMA-IR) and was calculated by using fasting glucose and insulin levels from measurements just before the start of the ipGTT by the following formula: HOMA-IR = glucose (mg/dl) × insulin (µU/ml)/405.

### Statistical analyses of phenotypic data

Time course data (body weight, blood glucose, and ipGTT) were analyzed by repeated measures two-way ANOVA (rANOVA). Weaning weights, areas under the curve (AUC) of ipGTTs as well as hormone measurements and HOMA-IR values were analyzed by *t* tests (unpaired, homoscedastic, two-tailed). If variances were significantly different between groups, *t* tests with Welch’s correction were performed. In all figures and in the text, the mean and standard deviation are given. Differences in body composition were addressed by analysis of covariance (ANCOVA), fat and lean mass were adjusted to the mean body weight of the (paternal) treatment group followed by *t* tests as described (Seyfarth et al. 2015). In all statistical tests, a *p* value below 0.05 was considered statistically significant. All analyses were performed using GraphPad Prism version 6.00 for Windows, GraphPad Software, La Jolla California, USA, www.graphpad.com.

### Double immunohistochemistry

Pancreata were fixed in neutral-buffered formalin for at least 24 h, embedded in paraffin, and cut in 1 µm sections for immunohistochemistry. Slides were stained by Roche-VENTANA Discovery^®^ XT automated slide stainer according to the company’s protocol. The following primary antibodies were used: polyclonal rabbit anti-human glucagon (1:1500; A0565, Dako) visualized by DAB (3,3′-diaminobenzidine) and polyclonal guinea pig anti-porcine insulin (1:750; A0564, Dako) stained with Fast Red. Heat antigen retrieval was followed by pretreatment of slides in Cell Conditioning Solution (CC1 mild, Ventana^®^, Roche). Counterstaining was done with hematoxylin. All sections were interpreted independently by two pathologists.

### Liver histology

For Oil-Red-O histochemistry liver samples were fixed in Baker’s formol calcium to preserve phospholipids. Tissue were frozen (−14 °C) and cut into 5 µm sections, rinsed with 60 % isopropanol, stained with freshly prepared Oil-Red-O (Merck Millipore, Cat. 105230) working solution for 10 min, and rinsed again in 60 % isopropanol. Counterstaining was performed with Mayer’s acid hemalaun and mounted with glycerol gelatin (Merck Millipore, Cat. 109242). All sections were independently evaluated by two expert pathologists.

### In vitro fertilization (IVF)

For the generation of the F1 generation by IVF, sperm was obtained from a total of three males per group (Dex, Veh) from the F0 generation. Oocytes were obtained from C57BL/6NTac mice, and embryos were transferred into CD-1 foster mothers. Sperm and oocyte isolation as well as IVF were conducted following standardized procedures described on the INFRAFRONTIER homepage. The whole procedure was carried out on three independent experimental days (always one male per group and experimental day).

### Sperm isolation for transcriptome analyses (F0 generation)

Sperm samples were collected after isoflurane anesthesia using the swim-up method as described (Brykczynska et al. 2010) with slight modifications. Briefly, dissected cauda epididymides from both sides were transferred into sperm motility medium (135 mM NaCl, 5 mM KCl, 1 mM MgSO_4_, 2 mM CaCl_2_, 30 mM Hepes pH 7.4; freshly supplemented with 10 mM lactate acid, 1 mM sodium pyruvate, 20 mg/ml BSA, and 25 mM NaHCO_3_), cut several times, and incubated at 37 °C for at least 20 min. The supernatant containing mature motile sperm was carefully removed and transferred into a new tube. To increase the yield, fresh sperm motility medium was added to the original tube (containing the epididymis and sperm), the tube was inverted to gently mix the sample, and centrifuged at 1000×*g* for 1 min. Afterward, the sample was again incubated at 37 °C for at least 20 min to allow motile sperm to swim to the surface, and the supernatant was carefully removed. The supernatants of both incubations were combined and centrifuged at 10,000×*g* for 1 min at 4 °C. The sperm pellet was frozen in liquid nitrogen.

### RNA isolation and transcriptome analyses

For RNA isolation, sperm samples were thawed on ice, washed 3 times with 500 µl phosphate-buffered saline (PBS) (centrifuged at 1000×*g* for 2 min, 4 °C in between washes), and the complete RNA was isolated using the miRNeasy Mini kit (Qiagen, Germany). The quality of the RNA was analyzed on the Bioanalyzer using the RNA 6000 pico Kit (Agilent, USA). Total RNA (about 10 ng) was amplified using the Ovation PicoSL WTA System V2 in combination with the Encore Biotin Module (Nugen). Amplified cDNA was hybridized on Affymetrix Mouse Gene 2.0 ST arrays containing about 34,000 probe sets. Staining and scanning (Scanner 3000 7G) were done according to the Affymetrix gene expression protocol including minor modifications as suggested in the Encore Biotin protocol.

Expression console (v.1.4.0.38, Affymetrix) was used for quality control and to obtain annotated normalized Robust Multi-array Average (RMA) gene-level data (standard settings including median polish and sketch-quantile normalization). Statistical analyses were performed by utilizing the statistical programming environment R (R Core Team 2014) implemented in CARMAweb (Rainer et al. 2006). Genewise testing for differential expression was done employing the limma *t* test and Benjamini-Hochberg multiple testing correction (FDR < 10 %). The cluster dendrogram was generated with the R script hclust. Array data have been submitted to GEO (GSE67290).

## Results

To test the hypothesis that altered plasma levels of insulin, leptin, and adiponectin are causative for EI of metabolic disorders in DIO models (Fullston et al. 2013; Ng et al. 2010, 2014; Wei et al. 2014), we induced such hormonal changes in the absence of obesity and most of its complication in F0 male mice by administration of dexamethasone. To focus on those hormonal changes in lean mice, we kept the F0 males on standard diet. The time frame from the start of the dexamethasone treatment to the start of the matings or sperm isolation was chosen to encompass the duration of more than one full cycle of spermatogenesis (40 days) (Adler 1996). This procedure ensured exposure of sperm to altered hormonal levels throughout spermatogenesis (Fig. 1).

### Effects of dexamethasone treatment in the F0 generation

#### Chronic dexamethasone treatment alters body composition

**Fig. 2:**
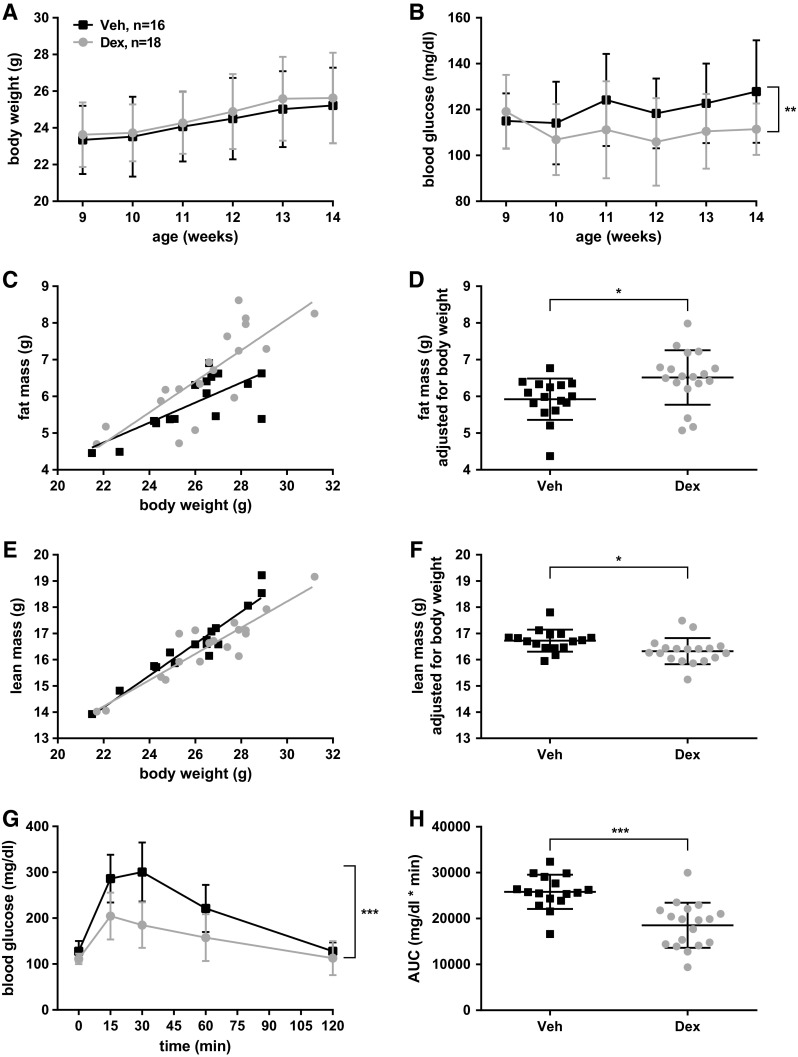
Chronic dexamethasone treatment alters body composition and glucose metabolism. C57BL/6NTac male mice were treated with 2 mg/kg dexamethasone (Dex: *n* = 18) or vehicle solution (Veh: *n* = 16). No body weight changes due to dexamethasone treatment were observed (**a**) but blood glucose levels were slightly reduced (**b**: rANOVA, *p* = 0.0026). Body composition was different at the end of the treatment with an increase in fat mass (**c**, **d**: *p* = 0.0141), and a decrease in lean mass (**e**, **f**: *p* = 0.0169) in the dexamethasone-treated group (**d**, **f**: ANCOVA analysis: fat mass and lean mass were adjusted to the mean body mass of the respective treatment group). Dexamethasone treatment improved glucose tolerance in ipGTT (G: rANOVA, *p* < 0.001; H: *t* test: *p* < 0.001, AUC: area under the curve). Mean ± standard deviation (**a**, **b**, **d**, **f**–**h**) or least square regression lines (**c**, **e**) are shown

Chronically elevated glucocorticoid levels in humans lead to central obesity in conjunction with a reduction of lean mass due to muscle atrophy (Kaur 2014; Vegiopoulos and Herzig 2007). To test whether we find similar alterations in our mouse model, we measured body weight during the course of the treatment and body composition at its end, at the age of 14 weeks. Although we did not detect any differences in body weight gain between the two groups (Fig. 2a), we found both a small but significant increase in fat mass (Fig. 2c, d) and a respective loss in lean mass in dexamethasone-treated male mice (Fig. 2e, f) in agreement with recent findings in a similar mouse model (Hochberg et al. 2015). Importantly, this increase in fat mass is more than one order of magnitude smaller than changes observed in BL6 mice on HFD [increase of about 0.5 g of adjusted fat mass in Dex F0 (Fig. 2d) as compared to an increase of more than 6 g of adjusted fat mass in HFD-fed F1 males (Supplementary Fig. 3B)]. Hence, although body composition is slightly altered, dexamethasone-treated male mice on standard diet are far from being obese.

#### Chronic dexamethasone treatment lowers blood glucose levels and improves glucose tolerance

Another hallmark of chronically elevated glucocorticoid levels are changes in glucose metabolism due to insulin resistance (IR) induced by glucocorticoid signaling in various tissues (Rafacho et al. 2014; Vegiopoulos and Herzig 2007). We measured fasting blood glucose concentrations during the course of the treatment and tested glucose tolerance at the end of the treatment. Surprisingly, and in contrast to most studies, we found a slight decrease in fasting blood glucose levels (Fig. 2b) as well as an increase in glucose tolerance following chronic dexamethasone treatment. In dexamethasone-treated male mice, the area under the curve (AUC) during ipGTT was reduced by approximately 25 % in comparison to vehicle-treated animals (Fig. 2g, h). In line with this finding, a recent study performed in rhesus macaques also reported strongly improved glucose tolerance following chronic dexamethasone treatment with a 50 % reduction of postprandial AUC (Cummings et al. 2013) presumably due to changes in blood insulin levels (see below).

#### Dexamethasone treatment recapitulates increases in insulin and leptin levels observed in DIO mouse models of EI

**Fig. 3:**
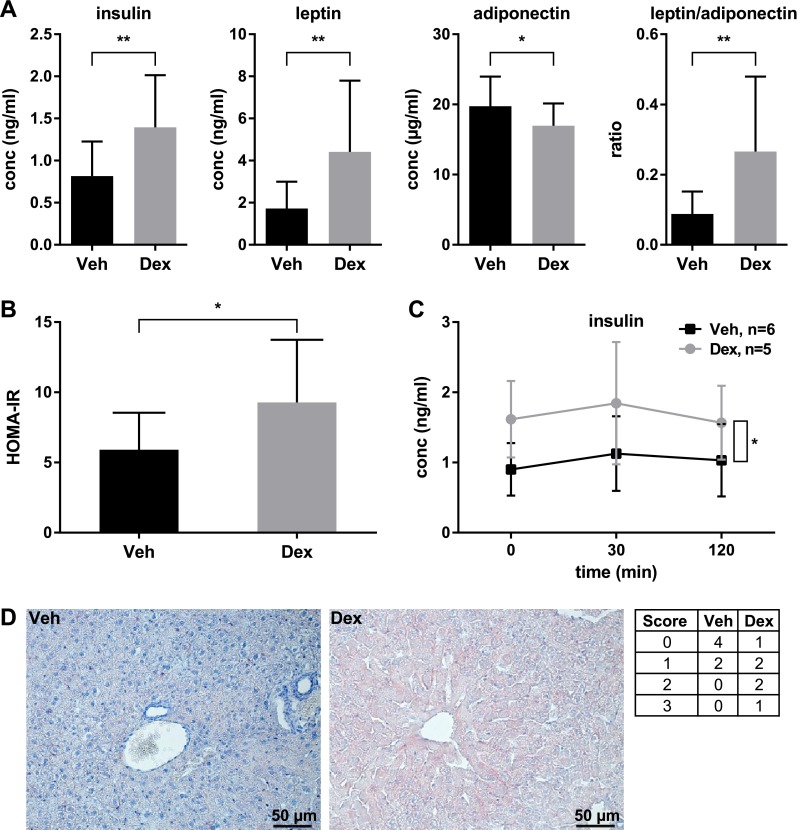
Chronic dexamethasone treatment alters insulin, leptin, and adiponectin plasma levels and pancreatic islet morphology. **a** Enzyme-linked immunosorbent assay (ELISA) measurement at the end of the dexamethasone treatment showed increases of insulin (*p* = 0.0044) and leptin (*p* = 0.0063) levels as well as a decrease in adiponectin (*p* = 0.0374) level. The leptin/adiponectin ratio was increased (*p* = 0.0039; *n* = 15–18). **b** Assessment of insulin sensitivity by HOMA-IR showed induction of modest insulin resistance by dexamethasone treatment (*p* = 0.0159, *n* = 15–17). **c** Insulin concentration during ipGTT is increased by dexamethasone treatment (rANOVA, *p* = 0.0474, *n* = 5–6). Mean ± standard deviation is shown. **d** Liver immunohistochemistry shows that liver fat content is increased in dexamethasone-treated animals. One representative sample is shown for each group and the scores of all samples analyzed (6 per group) are given in the table on the right. The higher the score, the more severe is the fat accumulation (0—none: no lipid staining, 1—mild: single cells with stained lipid, 2—moderate: patches of cells with stained lipid, and 3—distinct: most cells with stained lipid)

To confirm that our mouse model exhibits hormonal changes as those seen in obesity and MetS, we measured plasma levels of relevant hormones at the end of the dexamethasone treatment. Strikingly, in the dexamethasone-treated animals, both insulin (about 1.7-fold) and leptin (about 2.6-fold) were increased, whereas adiponectin was slightly decreased (about 0.86-fold) in comparison to vehicle-treated mice (Fig. 3a). Importantly, the leptin/adiponectin ratio was increased about threefold in the comparison of dexamethasone versus vehicle-treated male mice (Fig. 3a).

In C57BL/6NTac male mice, dexamethasone treatment induced only modest IR with a 1.6-fold increase in HOMA-IR (Fig. 3b), in line with an increase in liver fat content (Fig. 3d), which might explain the improved glucose tolerance that we observed (Fig. 2g, h). As glucose tolerance is influenced by both peripheral insulin sensitivity and blood insulin concentration, modest IR does not necessarily result in glucose intolerance if insulin levels increase accordingly. Thus, although being modestly insulin resistant (Fig. 3b), the significantly elevated insulin levels before and during ipGTT (Fig. 3a, c) in dexamethasone-treated male mice most likely over-compensate the glucocorticoid-induced IR. A similar overcompensation of IR by an altered hormonal response was also reported in the recent study in rhesus macaques mentioned above (Cummings et al. 2013), providing another example that hormonal adjustments can indeed be stronger than the relative resistance to them that is induced by dexamethasone treatment. Furthermore, as a result of dexamethasone treatment, pancreatic islets were increased in size, confluent and irregularly shaped (Supplementary Fig. S1) as reported in other rodent models [reviewed in (Rafacho et al. 2014)].

Taken together, the mouse model presented here specifically recapitulates key hormonal alterations of human obesity and MetS as well as DIO in mice. Importantly, the fold changes in insulin and leptin levels in our mouse model are in the same range as those in DIO mouse models of EI of metabolic phenotypes (Fullston et al. 2013; Wei et al. 2014), showing the validity of our approach to alter those hormone levels in the absence of obesity. Unfortunately, changes in adiponectin levels have not been addressed in the studies cited above.

### Effects of paternal dexamethasone treatment in the F1 generation

#### Paternal dexamethasone treatment does not influence metabolic parameters in the offspring via sperm

In order to unequivocally relate potential metabolic phenotypes in the offspring to epigenetic modifications present in sperm, we chose to generate offspring via IVF. The social interaction between male and female mice during the mating can cause a phenotype in the F1 generation via a mechanism called paternally induced maternal care (Curley et al. 2011; Mashoodh et al. 2012; Rando 2012). Here, the female judges the male and alters her behavior during pregnancy and lactation, which causes a phenotype in the offspring. Furthermore, factors present in seminal fluid have been suggested to be potential carriers of intergenerational information (Rando 2012). Hence, IVF is paramount to truly correlate an observed phenotype in the offspring to epigenetic alterations present in sperm, as it excludes both social interaction and seminal fluid as mechanisms of inheritance. We used sperm of 6 males [3 per treatment group (Veh, Dex)] of the F0 generation to generate offspring via IVF. Importantly, insulin (1.7-fold) and leptin (2.1-fold) (data not shown) levels were still increased to a similar extent at the time of sperm collection (1 week after the end of dexamethasone treatment) as they were at the end of the treatment (Fig. 3a). Thus, sperm used for IVF was exposed to increased insulin and leptin levels throughout spermatogenesis.

**Fig. 4:**
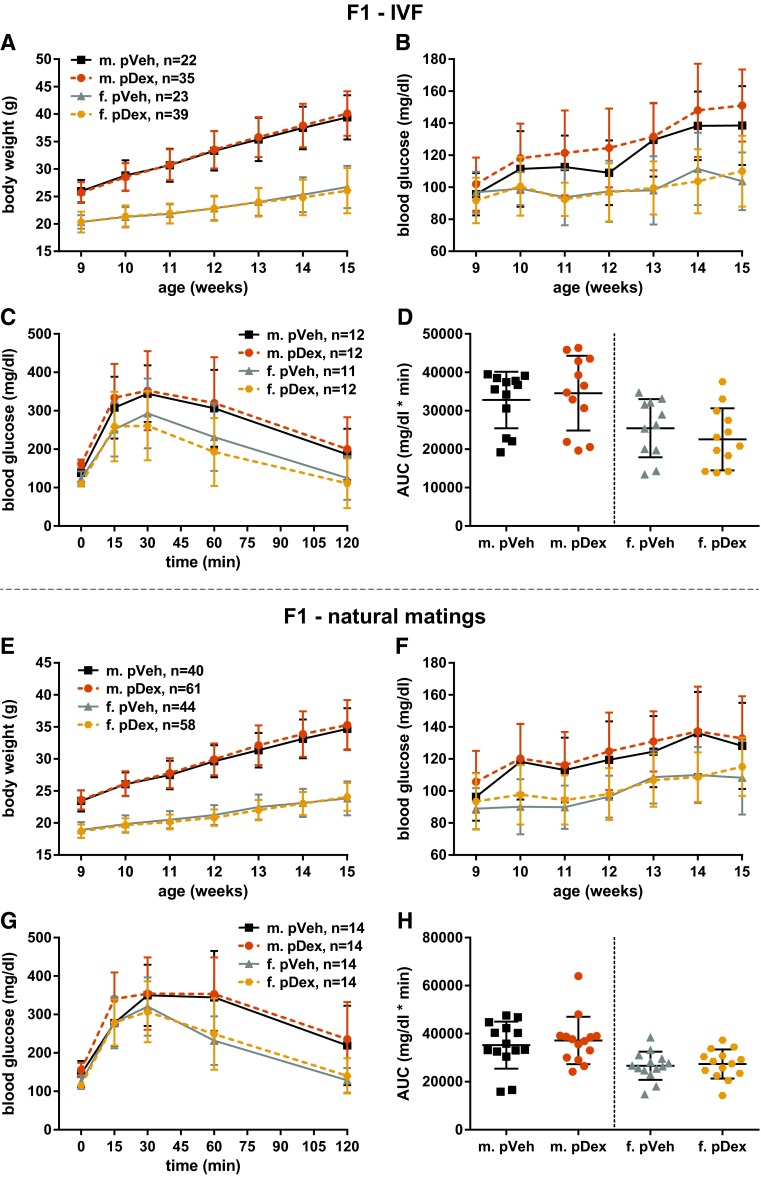
Paternal dexamethasone treatment does not alter the metabolic phenotype in the offspring. Offspring conceived either by IVF (**a**–**d**; **a**, **b**: *n* = 22–39, **c**, **d**: *n* = 11–12) or by natural mating (**e**–**h**; **e**, **f**: *n* = 40–61, **g**, **h**: *n* = 14) was fed a HFD starting at 9 weeks of age (m.: males, f.: females, pVeh: paternal Vehicle, pDex: paternal Dexamethasone). No differences due to paternal treatment were observed with respect to body weight gain (**a**, **e**), fasting blood glucose concentration (**b**, **f**) or glucose tolerance in an ipGTT (**c**, **d** and **g**, **h**). Mean ± standard deviation are shown

In offspring obtained via IVF, we did not find a difference due to paternal treatment in body weight at weaning (age of 3 weeks) neither for F1 males (paternal vehicle treatment (pVeh): 10.4 ± 1.6 g, pDex: 10.8 ± 1.6 g, *p* = 0.38) nor for F1 females (pVeh: 10.0 ± 1.8 g, pDex: 10.4 ± 1.7 g, *p* = 0.33). Thus, in order to enhance potentially subtle metabolic phenotypes, we challenged male and female mice of the F1 generation with a high-fat diet (HFD) starting at 9 weeks of age, a method successfully used in the field (Fullston et al. 2015). We analyzed the offspring for changes in body weight gain during the period of HFD challenge (Fig. 4a) and body composition at the age of 15 weeks (Figure S2). Male offspring of both paternal treatment groups weighed on average 26 g at the beginning of the HFD challenge and gained about 14 g during 6 weeks of HFD. F1 Females weighed around 20 g at the age of 9 weeks in both groups and gained about 6 g during the 6 week HFD challenge (Fig. 4a). In contrast to DIO mouse models that showed increased body weight in the F1 generation due to paternal HFD (Fullston et al. 2013, 2015), we could not detect any effect of paternal dexamethasone treatment on body weight at any time. Similarly, the measurement of body composition in male and female F1 animals did not provide evidence for differences depending on paternal treatment (Figure S2). This again is in contrast to findings in DIO mouse models that showed increased adiposity due to paternal HFD (Fullston et al. 2013, 2015).

Most studies focusing on EI of metabolic phenotypes reported striking alterations in glucose metabolism, especially in female offspring (Fullston et al. 2013; Ng et al. 2010, 2014; Wei et al. 2014). Accordingly, we measured fasting blood glucose levels during the HFD challenge (Fig. 4b) and glucose tolerance at the end of the HFD challenge in IVF-derived F1 mice (Fig. 4c, d). HFD challenge increased fasting blood glucose levels in males more dramatically than in females; however, we did not detect differences in fasting blood glucose levels due to paternal treatment (Fig. 4b). Alterations in F1 glucose tolerance due to paternal HFD have been reported in several studies, both in rat (Ng et al. 2010) and mouse (Fullston et al. 2013; Wei et al. 2014). We addressed glucose tolerance by ipGTT and found the expected sex-specific differences with females being more glucose tolerant than males (Oliveira et al. 2015); however, we did not detect any differences in glucose tolerance due to paternal dexamethasone treatment (Fig. 4c, d).

Taken together, the data from F1 offspring generated via IVF did not provide any evidence for EI via sperm, despite the fact that the changes in insulin and leptin levels in the dexamethasone-treated fathers are comparable to the changes in DIO mouse models (Fig. 3a).

#### Metabolic parameters are unchanged in offspring from natural matings

To our knowledge, all rodent studies performed thus far on EI of metabolic phenotypes via the paternal lineage were carried out by natural mating of dietary-challenged males with untreated females. Yet, one study on the EI of stress-related behavioral phenotypes compared offspring conceived by natural mating or IVF and found an inherited phenotype only if the offspring was conceived naturally, arguing for inheritance independent from sperm (Dietz et al. 2011). To test whether the absence of a detectable metabolic phenotype in the offspring generated by IVF is due to the lack of social interaction during mating or lack of factors present in seminal fluid, we also generated offspring by natural fecundation. A total of 13 males (6 Veh, 7 Dex) of the F0 generation went into natural matings and all of them generated offspring. Like for the IVF experiments described above, in the beginning of the natural matings (1 week after dexamethasone treatment), insulin and leptin levels were still in the same range as at the end of the treatment (Fig. 3a and see above).

Also in the F1 generation from natural matings, we did not detect differences in weaning weight depending on the paternal treatment for males (pVeh: 9.7 ± 1.9 g, pDex: 9.8 ± 1.5 g, *p* = 0.75) and females (pVeh: 9.4 ± 1.5 g, pDex: 9.4 ± 1.4 g, *p* = 0.93). At the start of the HFD (age of 9 weeks), the average body weight was similar in both groups (males: 23–24 g, females: 18–19 g) and body weight increased more for males (about 11–12 g) than for females (about 5–6 g) during the HFD challenge. Again, we did not detect differences in body weight gain due to paternal dexamethasone treatment (Fig. 4e). Consistent with these findings, we did not find evidence for alterations in body composition due to paternal treatment in the F1 generation from natural matings (Figure S3). As offspring from natural matings was not generated at a precisely defined time point (as in IVF) and as the effects induced by the hormonal changes due to dexamethasone treatment might be transient, we compared animals with regard to the round of mating they were sired in. We did, however, not find any differences in body weight gain dependent on how long after the treatment they were sired (data not shown).

We did not find difference due to paternal dexamethasone treatment in fasting blood glucose levels in F1 males and females during the HFD challenge (Fig. 4f). Also, ipGTTs did not provide evidence for differences in glucose tolerance caused by paternal dexamethasone treatment (Fig. 4g, h).

Taken together, also generating offspring via natural matings did not lead to metabolic differences in offspring due to paternal dexamethasone treatment. Hence, the changes in plasma levels of insulin, leptin, and adiponectin as well as the dexamethasone treatment as such did not lead to intergenerational inheritance of metabolic phenotypes such as body weight, body composition, or glucose tolerance.

#### Dexamethasone treatment does not alter sperm transcriptome

Although we did not find phenotypic differences in the F1 generation, we analyzed whether the paternal dexamethasone treatment leads to alterations in sperm transcriptomes. Changes in sperm transcriptomes might provide initial evidence toward traits other than the metabolic phenotypes addressed here that could be altered in the offspring. However, microarray-based transcriptome analyses did not show any significant change (criteria: FDR < 10 %, FC > 1.2) due to the treatment (the complete transcriptome data are available from GEO under accession ID GSE67290). Also, hierarchical cluster (HCL) analysis of genome-wide expression profiles did not separate the dexamethasone and vehicle-treated groups (Figure S4). However, we cannot exclude that other epigenetic changes, such as miRNAs, DNA methylation, or histone modifications, could be carriers of information between generations due to dexamethasone treatment and the accompanying hormonal alterations.

## Discussion

Several studies have reported examples of EI for various traits in mammals (Heard and Martienssen 2014; Nadeau 2009, 2015; Rando 2012; Rando and Simmons 2015; Somer and Thummel 2014; Szyf 2015). In particular, an important role of the paternal diet for the metabolic phenotype of the next generation has been established (Carone et al. 2010; Fullston et al. 2013; Ng et al. 2010, 2014; Wei et al. 2014). Although these studies did not perform IVF to unequivocally show inheritance via the gametes, some of them reported alterations of sperm DNA methylation and miRNA content (Carone et al. 2010; Fullston et al. 2013; Ng et al. 2010; Radford et al. 2014; Wei et al. 2014). Alterations of the sperm epigenome have also been reported for other environmental factors such as stress (Dias and Ressler 2014; Rodgers et al. 2013), cocaine (Vassoler et al. 2013), and the hepatotoxin carbon tetrachloride (Zeybel et al. 2012), demonstrating that the sperm epigenome is receptive for environmental stimuli. One important step toward understanding EI of MDs is to decipher the mechanism(s) and specific factors of soma-to-germline information transfer. Hormones and circulating RNAs have been suggested as prime candidates for such factors (Jablonka and Raz 2009; Sharma 2013; Szyf 2015).

Importantly, the data presented in our study demonstrate that altered plasma levels of insulin, leptin, and adiponectin in a physiological range that is also observed in DIO mouse models (Fullston et al. 2013; Ng et al. 2010, 2014; Wei et al. 2014) are not sufficient for the EI of metabolic phenotypes, such as body weight, body composition, and glucose homeostasis, via the paternal lineage even when the progeny is challenged with a HFD. Moreover, our mouse model also does not point toward a role for elevated glucocorticoid levels in EI of altered metabolism across generations.

MDs such as obesity, MetS, and T2D are characterized by a number of altered factors besides the hormones analyzed in our study that could be implicated in EI. Although our study suggests that altered levels of insulin, leptin, and adiponectin are not sufficient for soma-to-germline information transfer, this does not exclude the possibility that they—alone or in combination—are required for this function in conjunction with other factor(s). Further studies analyzing additional candidate factors in isolation or in combination with those analyzed here, will be necessary to identify the factors required and/or sufficient to confer soma-to-germline information transfer in EI of metabolic phenotypes.

## Electronic supplementary material

Supplementary Figure S1: Dexamethasone treatment alters pancreatic islet morphology. Pancreas immunohistochemistry shows that pancreatic islets are increased in size, confluent and irregularly shaped (compare top left and right) and that glucagon producing α-cells tend to centralize (bottom right, arrows) in dexamethasone-treated animals. Supplementary material 1 (PDF 4244 kb)

Supplementary Figure S2: Paternal dexamethasone treatment does not alter body composition in the offspring generated by IVF. Offspring conceived by IVF (n = 22-39) were fed a HFD for 6 weeks, starting at 9 weeks of age (m.: males (A-D), f.: females (E–H), pVeh: paternal Vehicle, pDex: paternal Dexamethasone). No differences in body composition were observed (B, D, F, H: ANCOVA analysis: fat mass and lean mass were adjusted to the mean body mass of the respective treatment group). Least square regression lines (A, C, E, G) or mean ± standard deviation (B, D, F, H) are shown. Supplementary material 2 (PDF 241 kb)

Supplementary Figure S3: Paternal dexamethasone treatment does not alter body composition in the offspring generated by natural matings. Offspring conceived naturally (n = 40-61) were fed a HFD for 6 weeks, starting at 9 weeks of age (m.: males (A-D), f.: females (E–H), pVeh: paternal Vehicle, pDex: paternal Dexamethasone). No differences in body composition were observed (B, D, F, H: ANCOVA analysis: fat mass and lean mass were adjusted to the mean body mass of the respective treatment group). Least square regression lines (A, C, E, G) or mean ± standard deviation (B, D, F, H) are shown. Supplementary material 3 (PDF 273 kb)

Supplementary Figure S4: Chronic dexamethasone treatment does not alter sperm transcriptome. Hierarchical cluster (HCL) analysis of sperm transcriptomes does not show separation of the two treatment groups. The y-axis (height) represents the distance between samples. A total of 15 sperm samples (Veh: n = 7, Dex: n = 8) were analyzed on Affymetrix Mouse Gene ST 2.0 arrays. Supplementary material 4 (PDF 157 kb)

## References

[CR1] Adler ID (1996). Comparison of the duration of spermatogenesis between male rodents and humans. Mutat Res.

[CR2] Ahn SW, Gang GT, Kim YD, Ahn RS, Harris RA (2013). Insulin directly regulates steroidogenesis via induction of the orphan nuclear receptor DAX-1 in testicular Leydig cells. J Biol Chem.

[CR3] Binder NK, Sheedy JR, Hannan NJ, Gardner DK (2015). Male obesity is associated with changed spermatozoa Cox4i1 mRNA level and altered seminal vesicle fluid composition in a mouse model. Mol Hum Reprod.

[CR4] Bluher M (2014). Adipokines - removing road blocks to obesity and diabetes therapy. Mol Metab.

[CR5] Bromfield JJ (2014). Seminal fluid and reproduction: much more than previously thought. J Assist Reprod Genet.

[CR6] Bromfield JJ, Schjenken JE, Chin PY, Care AS, Jasper MJ (2014). Maternal tract factors contribute to paternal seminal fluid impact on metabolic phenotype in offspring. Proc Natl Acad Sci U S A.

[CR7] Brykczynska U, Hisano M, Erkek S, Ramos L, Oakeley EJ (2010). Repressive and active histone methylation mark distinct promoters in human and mouse spermatozoa. Nat Struct Mol Biol.

[CR8] Carone BR, Fauquier L, Habib N, Shea JM, Hart CE (2010). Paternally induced transgenerational environmental reprogramming of metabolic gene expression in mammals. Cell.

[CR9] Cummings BP, Bremer AA, Kieffer TJ, D’Alessio D, Havel PJ (2013). Investigation of the mechanisms contributing to the compensatory increase in insulin secretion during dexamethasone-induced insulin resistance in rhesus macaques. J Endocrinol.

[CR10] Curley JP, Mashoodh R, Champagne FA (2011). Epigenetics and the origins of paternal effects. Horm Behav.

[CR11] Desai M, Jellyman JK, Ross MG (2015). Epigenomics, gestational programming and risk of metabolic syndrome. Int J Obes (Lond).

[CR12] Dias BG, Ressler KJ (2014). Parental olfactory experience influences behavior and neural structure in subsequent generations. Nat Neurosci.

[CR13] Dietz DM, Laplant Q, Watts EL, Hodes GE, Russo SJ (2011). Paternal transmission of stress-induced pathologies. Biol Psychiatry.

[CR14] Falahi E, Khalkhali Rad AH, Roosta S (2013). What is the best biomarker for metabolic syndrome diagnosis?. Diabetes Metab Syndr.

[CR15] Farag YM, Gaballa MR (2011). Diabesity: an overview of a rising epidemic. Nephrol Dial Transplant.

[CR16] Fasshauer M, Klein J, Neumann S, Eszlinger M, Paschke R (2002). Hormonal regulation of adiponectin gene expression in 3T3-L1 adipocytes. Biochem Biophys Res Commun.

[CR17] Fullston T, Ohlsson Teague EM, Palmer NO, DeBlasio MJ, Mitchell M (2013). Paternal obesity initiates metabolic disturbances in two generations of mice with incomplete penetrance to the F2 generation and alters the transcriptional profile of testis and sperm microRNA content. FASEB J.

[CR18] Fullston T, McPherson NO, Owens JA, Kang WX, Sandeman LY (2015). Paternal obesity induces metabolic and sperm disturbances in male offspring that are exacerbated by their exposure to an “obesogenic” diet. Physiol Rep.

[CR19] Hara K, Shojima N, Hosoe J, Kadowaki T (2014). Genetic architecture of type 2 diabetes. Biochem Biophys Res Commun.

[CR20] Heard E, Martienssen RA (2014). Transgenerational epigenetic inheritance: myths and mechanisms. Cell.

[CR21] Heinz JF, Singh SP, Janowitz U, Hoelker M, Tesfaye D (2015). Characterization of adiponectin concentrations and molecular weight forms in serum, seminal plasma, and ovarian follicular fluid from cattle. Theriogenology.

[CR22] Hirschhorn JN, Gajdos ZK (2011). Genome-wide association studies: results from the first few years and potential implications for clinical medicine. Annu Rev Med.

[CR23] Hochberg I, Harvey I, Tran QT, Stephenson EJ, Barkan AL (2015). Gene expression changes in subcutaneous adipose tissue due to Cushing’s disease. J Mol Endocrinol.

[CR24] Jablonka E, Raz G (2009). Transgenerational epigenetic inheritance: prevalence, mechanisms, and implications for the study of heredity and evolution. Q Rev Biol.

[CR25] Kaur J (2014). A comprehensive review on metabolic syndrome. Cardiol Res Prac.

[CR26] Locke AE, Kahali B, Berndt SI, Justice AE, Pers TH (2015). Genetic studies of body mass index yield new insights for obesity biology. Nature.

[CR27] Mashoodh R, Franks B, Curley JP, Champagne FA (2012). Paternal social enrichment effects on maternal behavior and offspring growth. Proc Natl Acad Sci U S A.

[CR28] Nadeau JH (2009). Transgenerational genetic effects on phenotypic variation and disease risk. Hum Mol Genet.

[CR29] Nadeau JH (2015). The nature of evidence for and against epigenetic inheritance. Genome Biol.

[CR30] Ng SF, Lin RC, Laybutt DR, Barres R, Owens JA (2010). Chronic high-fat diet in fathers programs beta-cell dysfunction in female rat offspring. Nature.

[CR31] Ng SF, Lin RC, Maloney CA, Youngson NA, Owens JA (2014). Paternal high-fat diet consumption induces common changes in the transcriptomes of retroperitoneal adipose and pancreatic islet tissues in female rat offspring. FASEB J.

[CR32] Oliveira RB, Maschio DA, Carvalho CP, Collares-Buzato CB (2015). Influence of gender and time diet exposure on endocrine pancreas remodeling in response to high fat diet-induced metabolic disturbances in mice. Ann Anat.

[CR33] Rabe K, Lehrke M, Parhofer KG, Broedl UC (2008). Adipokines and insulin resistance. Mol Med.

[CR34] Radford EJ, Ito M, Shi H, Corish JA, Yamazawa K (2014). In utero effects. In utero undernourishment perturbs the adult sperm methylome and intergenerational metabolism. Science.

[CR35] Rafacho A, Ortsater H, Nadal A, Quesada I (2014). Glucocorticoid treatment and endocrine pancreas function: implications for glucose homeostasis, insulin resistance and diabetes. J Endocrinol.

[CR36] Rainer J, Sanchez-Cabo F, Stocker G, Sturn A, Trajanoski Z (2006). CARMAweb: comprehensive R- and bioconductor-based web service for microarray data analysis. Nucleic Acids Res.

[CR37] Ramachandran R, Maddineni S, Ocon-Grove O, Hendricks G, Vasilatos-Younken R (2013). Expression of adiponectin and its receptors in avian species. Gen Comp Endocrinol.

[CR38] Rando OJ (2012). Daddy issues: paternal effects on phenotype. Cell.

[CR39] Rando OJ, Simmons RA (2015). I’m eating for two: parental dietary effects on offspring metabolism. Cell.

[CR40] R Core Team (2014) R: A language and environment for statistical computing

[CR41] Rodgers AB, Morgan CP, Bronson SL, Revello S, Bale TL (2013). Paternal stress exposure alters sperm microRNA content and reprograms offspring HPA stress axis regulation. J Neurosci.

[CR42] Satoh N, Naruse M, Usui T, Tagami T, Suganami T (2004). Leptin-to-adiponectin ratio as a potential atherogenic index in obese type 2 diabetic patients. Diabetes Care.

[CR43] Seyfarth K, Poschmann G, Rozman J, Fromme T, Rink N (2015). The development of diet-induced obesity and associated metabolic impairments in Dj-1 deficient mice. J Nutr Biochem.

[CR44] Sharma A (2013). Transgenerational epigenetic inheritance: focus on soma to germline information transfer. Prog Biophys Mol Biol.

[CR45] Shimizu H, Oh IS, Okada S, Mori M (2007). Leptin resistance and obesity. Endocr J.

[CR46] Slieker LJ, Sloop KW, Surface PL, Kriauciunas A, LaQuier F (1996). Regulation of expression of ob mRNA and protein by glucocorticoids and cAMP. J Biol Chem.

[CR47] Somer RA, Thummel CS (2014). Epigenetic inheritance of metabolic state. Curr Opin Genet Dev.

[CR48] Stoger R (2008). The thrifty epigenotype: an acquired and heritable predisposition for obesity and diabetes?. Bioessays.

[CR49] Szyf M (2015). Nongenetic inheritance and transgenerational epigenetics. Trends Mol Med.

[CR50] Vassoler FM, White SL, Schmidt HD, Sadri-Vakili G, Pierce RC (2013). Epigenetic inheritance of a cocaine-resistance phenotype. Nat Neurosci.

[CR51] Vegiopoulos A, Herzig S (2007). Glucocorticoids, metabolism and metabolic diseases. Mol Cell Endocrinol.

[CR52] Wang G, Guo Y, Zhou T, Shi X, Yu J (2013). In-depth proteomic analysis of the human sperm reveals complex protein compositions. J Proteomics.

[CR53] Wei Y, Yang CR, Wei YP, Zhao ZA, Hou Y (2014). Paternally induced transgenerational inheritance of susceptibility to diabetes in mammals. Proc Natl Acad Sci U S A.

[CR54] Williams L, Seki Y, Vuguin PM, Charron MJ (2014). Animal models of in utero exposure to a high fat diet: a review. Biochim Biophys Acta.

[CR55] Zamorano PL, Mahesh VB, De Sevilla LM, Chorich LP, Bhat GK (1997). Expression and localization of the leptin receptor in endocrine and neuroendocrine tissues of the rat. Neuroendocrinology.

[CR56] Zeybel M, Hardy T, Wong YK, Mathers JC, Fox CR (2012). Multigenerational epigenetic adaptation of the hepatic wound-healing response. Nat Med.

