# Supplementary figures and images for: Dexamethasone treatment alters insulin, leptin, and adiponectin levels in male mice as observed in DIO but does not lead to alterations of metabolic phenotypes in the offspring
Source: Mamm Genome. 2015 Dec 11;27:17–28. doi: 10.1007/s00335-015-9616-5 (PMC4731435; doi:10.1007/s00335-015-9616-5)

## Supplementary Figure S1

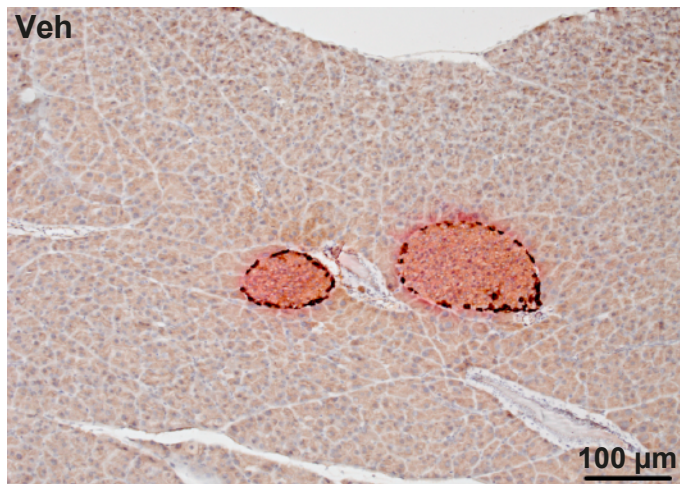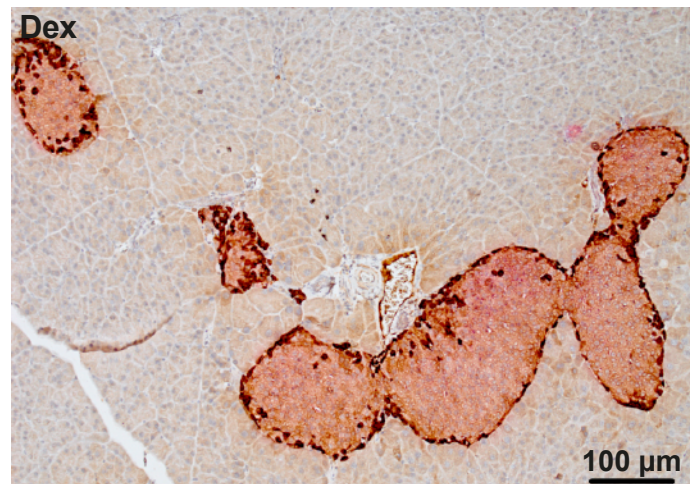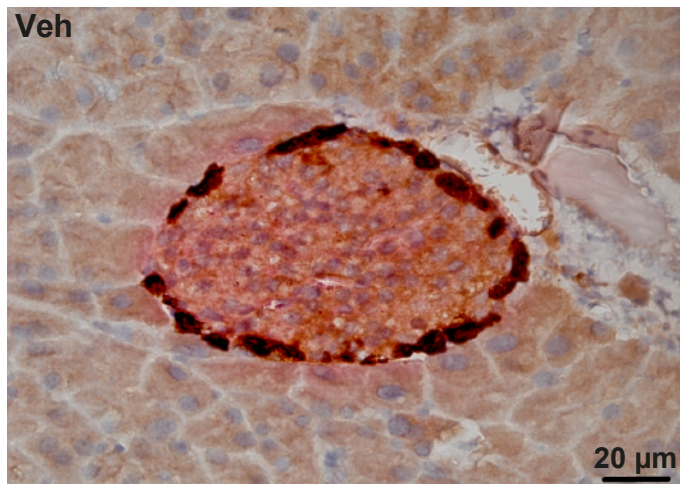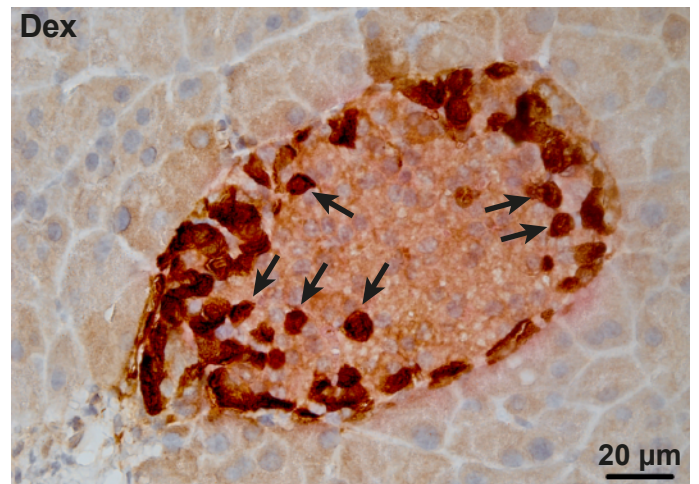

Supplement: Supplementary file 1 — Supplementary Figure S1: Dexamethasone treatment alters pancreatic islet morphology. Pancreas immunohistochemistry shows that pancreatic islets are increased in size, confluent and irregularly shaped (compare top left and right) and that glucagon producing α-cells tend to centralize (bottom right, arrows) in dexamethasone-treated animals. Supplementary material 1 (PDF 4244 kb) [file 335_2015_9616_MOESM1_ESM.pdf]

# Supplementary Figure S2

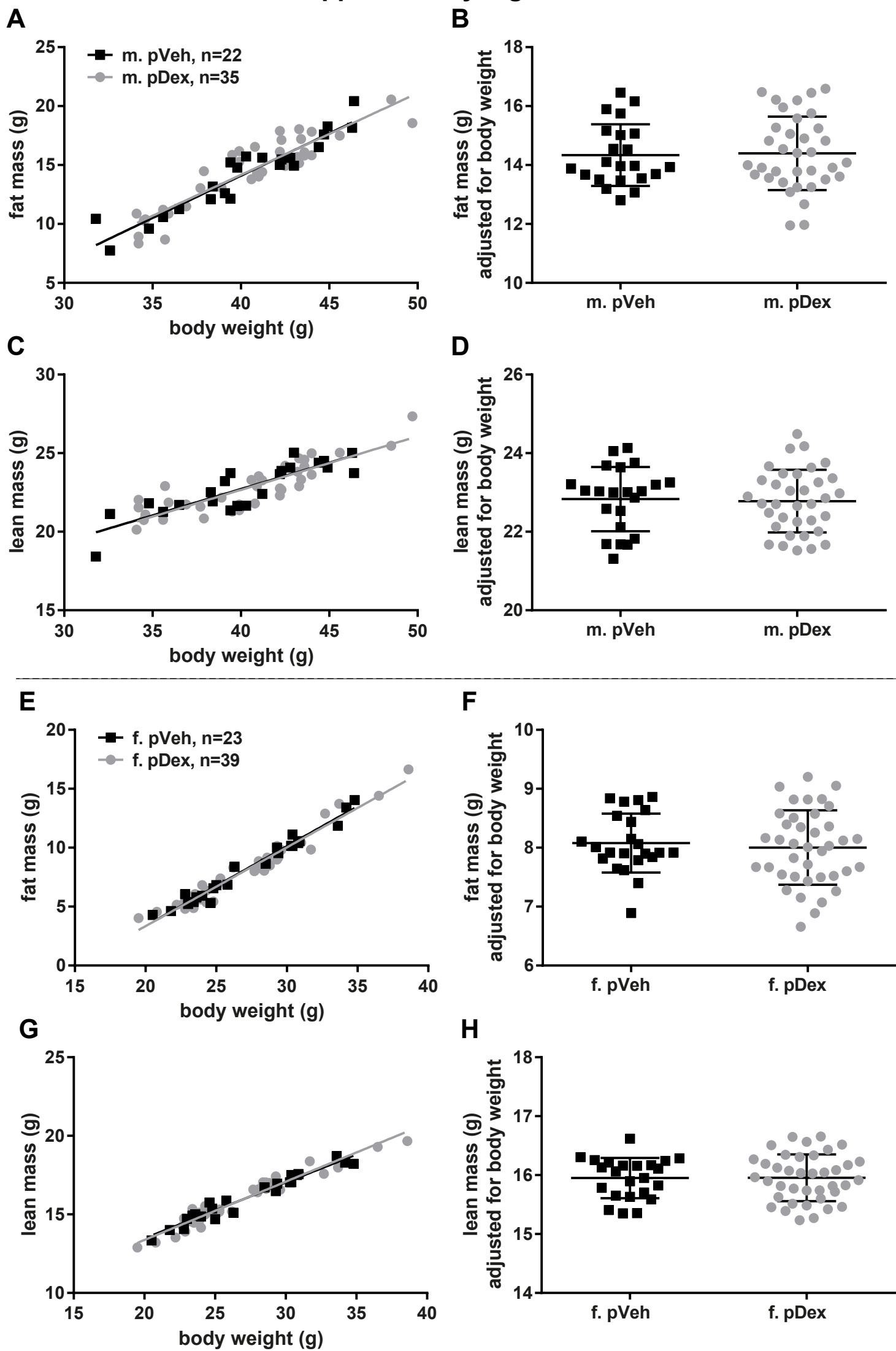

Supplement: Supplementary file 2 — Supplementary Figure S2: Paternal dexamethasone treatment does not alter body composition in the offspring generated by IVF. Offspring conceived by IVF (n = 22-39) were fed a HFD for 6 weeks, starting at 9 weeks of age (m.: males (A-D), f.: females (E–H), pVeh: paternal Vehicle, pDex: paternal Dexamethasone). No differences in body composition were observed (B, D, F, H: ANCOVA analysis: fat mass and lean mass were adjusted to the mean body mass of the respective treatment group). Least square regression lines (A, C, E, G) or mean ± standard deviation (B, D, F, H) are shown. Supplementary material 2 (PDF 241 kb) [file 335_2015_9616_MOESM2_ESM.pdf]

# Supplementary Figure S3

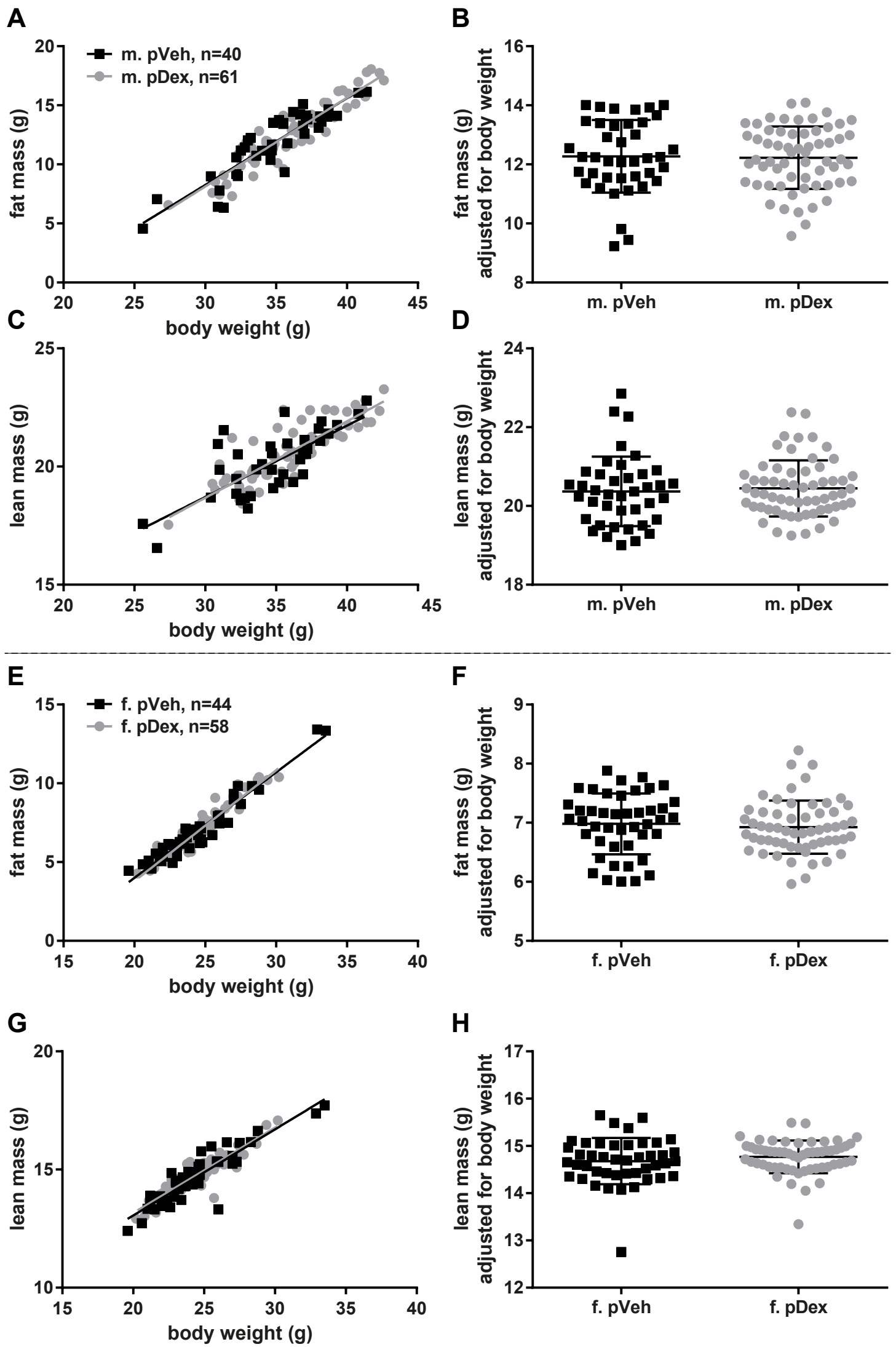

Supplement: Supplementary file 3 — Supplementary Figure S3: Paternal dexamethasone treatment does not alter body composition in the offspring generated by natural matings. Offspring conceived naturally (n = 40-61) were fed a HFD for 6 weeks, starting at 9 weeks of age (m.: males (A-D), f.: females (E–H), pVeh: paternal Vehicle, pDex: paternal Dexamethasone). No differences in body composition were observed (B, D, F, H: ANCOVA analysis: fat mass and lean mass were adjusted to the mean body mass of the respective treatment group). Least square regression lines (A, C, E, G) or mean ± standard deviation (B, D, F, H) are shown. Supplementary material 3 (PDF 273 kb) [file 335_2015_9616_MOESM3_ESM.pdf]

Supplementary Figure S4

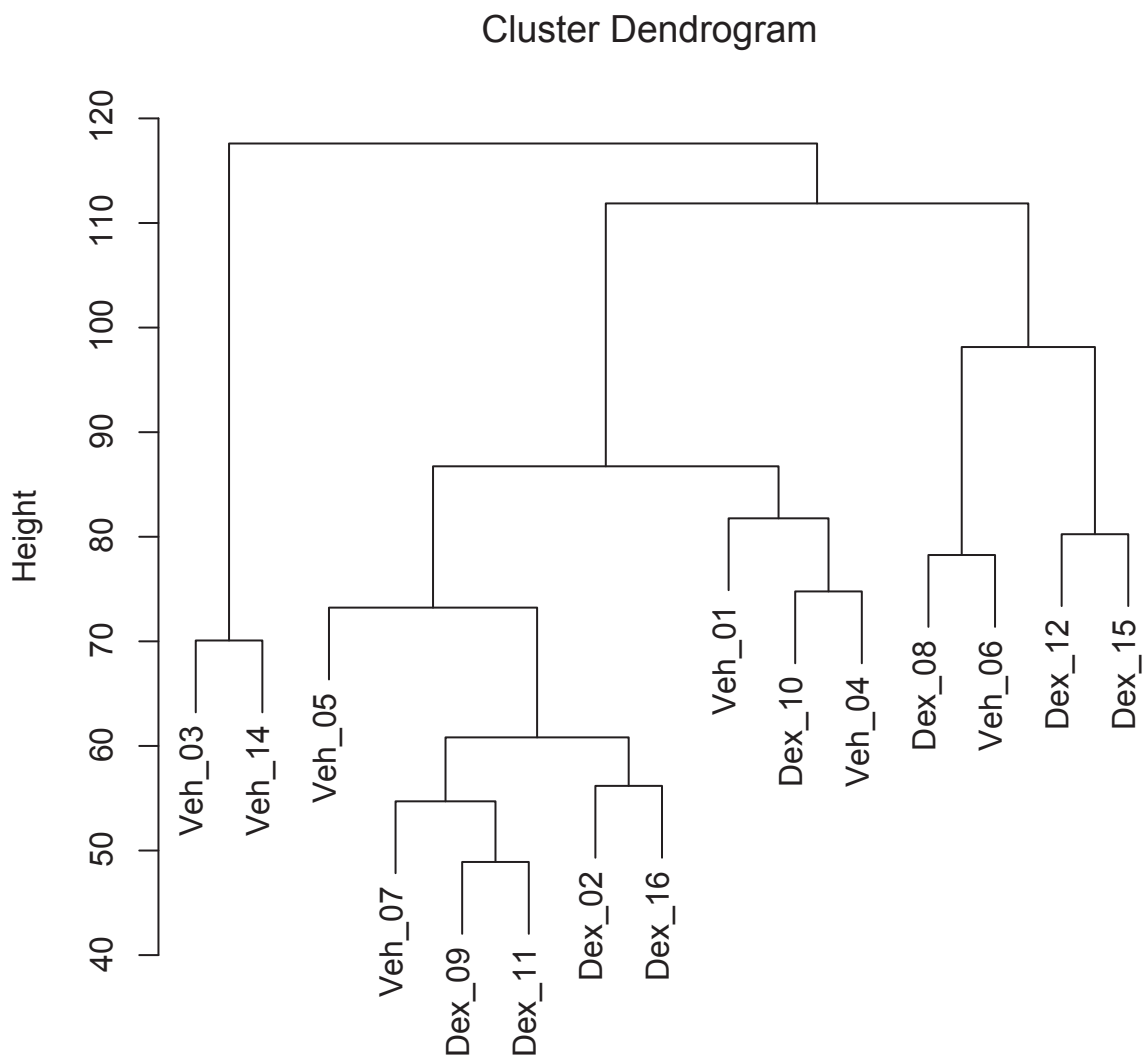

Supplement: Supplementary file 4 — Supplementary Figure S4: Chronic dexamethasone treatment does not alter sperm transcriptome. Hierarchical cluster (HCL) analysis of sperm transcriptomes does not show separation of the two treatment groups. The y-axis (height) represents the distance between samples. A total of 15 sperm samples (Veh: n = 7, Dex: n = 8) were analyzed on Affymetrix Mouse Gene ST 2.0 arrays. Supplementary material 4 (PDF 157 kb) [file 335_2015_9616_MOESM4_ESM.pdf]
